# Is There a Role for Sodium Fluoride in Otosclerosis Treatment?

**DOI:** 10.1002/lary.70137

**Published:** 2025-11-05

**Authors:** Timothy Shim, Kevin Wong, Michael J. Ruckenstein

**Affiliations:** ^1^ Department of Otorhinolaryngology University of Pennsylvania Philadelphia Pennsylvania USA

**Keywords:** hearing loss, otosclerosis, otospongiosis, sodium fluoride, stapedectomy, stapedotomy

## Abstract

Various reports have investigated the role of NaF for stabilizing SNHL in the setting of otosclerosis; however, there currently remains no established practice guideline or widespread consensus. The purpose of this paper is to interpret the literature to determine best practices for the use of NaF in otosclerosis management.

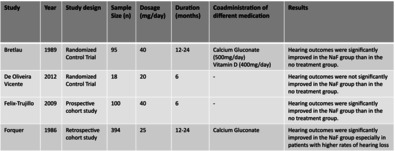

## 1 Background

Otosclerosis is a disease characterized by the resorption of otic capsule bone and subsequent disorganized redeposition. This disease is classically associated with conductive hearing loss, however, sensorineural hearing loss (SNHL) can also be seen in advanced cases due to enzymatic changes within the inner ear from bony metabolism. Sodium fluoride (NaF) is a well‐established enzymogenetic inhibitor and has been theorized to inhibit cytotoxic enzymes that accumulate within the perilymph of patients with otosclerosis [1].

Scattered reports have investigated the role of NaF for stabilizing SNHL in the setting of otosclerosis, however, there currently remains no established practice guideline or widespread consensus. The purpose of this paper is to interpret the literature to determine best practice for the use of NaF in otosclerosis management.

## 2 Literature Review

Contemporary management of otosclerosis may include medical or surgical management. Surgical management includes stapedectomy, stapedotomy, and implantable bone conduction devices. Conservative management may involve observation or hearing aids.

Forquer et al. published one of the earliest large‐scale series of 192 otosclerosis patients treated with NaF (Table 1) [1]. Results showed that NaF slowed or halted SNHL in 63% of patients with cochlear otosclerosis and in 46% of patients with stapedial otosclerosis. Patients with a greater rate of pre‐treatment hearing loss were more likely to benefit from NaF, suggesting that active otospongiotic processes may be more responsive to NaF. This would further support the enzymatic theory of disease.

**TABLE 1:** Selected investigations on the usage of sodium fluoride (NaF) in otosclerosis treatment.

Study	Year	Study design	Sample size (n)	Dosage (mg/day)	Duration (months)	Coadministration of different medication	Results
Bretlau	1989	Randomized control trial	95	40	12–24	Calcium gluconate (500 mg/day) Vitamin D (400 mg/day)	Hearing outcomes were significantly improved in the NaF group than in the no treatment group.
De Oliveira Vicente	2012	Randomized control trial	18	20	6	—	Hearing outcomes were not significantly improved in the NaF group than in the no treatment group.
Felix‐Trujillo	2009	Prospective cohort study	100	40	6	—	Hearing outcomes were significantly improved in the NaF group than in the no treatment group.
Forquer	1986	Retrospective cohort study	394	25	12–24	Calcium gluconate	Hearing outcomes were significantly improved in the NaF group especially in patients with higher rates of hearing loss

Felix‐Trujillo et al. published a nonrandomized, double‐blinded study on 50 otosclerosis patients who underwent NaF treatment (40 mg/day) matched to an otosclerosis cohort of 50 patients that did not receive treatment [2]. Both groups were treated with NaF for 6 months and then underwent stapedectomy. The authors found greater post‐treatment hearing improvement in the NaF group than the control group.

In a systematic review by Hentschel et al., the authors found only weak evidence to justify the efficacy of NaF in preventing hearing deterioration [3]. Among nine included articles, only two studies provided high‐quality evidence appropriate for further review. Bretlau et al. reported a greater rate of hearing deterioration in the placebo group compared to the NaF treatment group with an absolute risk reduction of 18% [4]. In contrast, the second study of the two by Oliveria Vicente et al. found no significant difference in hearing outcomes [5]. Bias from study design, small sample sizes, and differences in study factors including dosage and duration of treatment, comedications, outcome measure choices, and follow‐up duration may explain discrepancies in results. For example, one study did not measure high pure‐tone audiometric frequencies [4] and the other had follow‐up duration of less than a year [5]. Neither study adequately randomized treatment assignments nor blinded participants; thus, introduced bias can potentially skew the effects of studied interventions.

Additionally, clinicians that utilize NaF should remain alert to adverse side effects such as allergic dermatitis, arthritic symptoms, hair loss, and changes in dental enamel.^**3**^ More serious complications of long‐term fluoride supplementation are well described in the literature and include fluorosis—characterized by changes in bone structure and skeletal fragility, neurotoxicity, and alterations in hepatic and renal function. Other literature suggests fluoride supplementation has adverse cardiometabolic health implications and is linked to lipid disturbances. On a cellular level, it has been associated with increased oxidative stress causing DNA damage. Chronic exposure to fluoride above permissible limits in drinking water (typically 1.5 ppm) can lead to fluorosis with thresholds even lower in pediatric populations. Studies that evaluated otosclerosis patients utilized fluoride doses several orders greater in magnitude.

### 2.1 Future Directions

Today, there is no standardized approach to the inclusion of NaF for the management of otosclerosis. There exists a need for higher quality evidence to support any audiologic benefits and safety of NaF in otosclerosis. Larger, randomized multi‐center investigations with standardized treatment protocols have the potential to provide robust data in the future to guide selective NaF use. Even so, such clinical trials would be intensive while exposing patients with other rehabilitative options to potential risks of NaF.

## 3 Best Practice

For conservative medical management of otosclerosis, NaF remains a non‐operative treatment alternative that has the potential to ameliorate rates of hearing degradation. However, there is only a low level of evidence that NaF is an effective treatment, with many investigations demonstrating the toxicity of chronic fluoride supplementation. Thus, while NaF in conjunction with hearing aids may stabilize and restore some degree of hearing while presenting an attractive alternative to surgery, careful consideration of long‐term NaF supplementation is essential for safe medical management. In the age of cochlear implants, the need to subject patients to long‐term and potentially toxic medication must be questioned.

## 4 Level of Evidence

One systematic review of the literature (level 1), two randomized control trials (level 1), one prospective cohort study (level 2), and one retrospective cohort study were evaluated (level 4).

## Conflicts of Interest

The authors declare no conflicts of interest.
